# High mutual cooperation rates in rats learning reciprocal altruism: The role of payoff matrix

**DOI:** 10.1371/journal.pone.0204837

**Published:** 2019-01-02

**Authors:** Guillermo E. Delmas, Sergio E. Lew, B. Silvano Zanutto

**Affiliations:** 1 Universidad de Buenos, Facultad de Ingeniería, Instituto de Ingeniería Biomédica, Aires, Buenos Aires, Argentina; 2 Instituto de Biología y Medicina Experimental (IBYME-CONICET), Laboratorio de Biología del Comportamiento, Ciudad de Buenos Aires, Buenos Aires, Argentina; Southwest University, CHINA

## Abstract

Cooperation is one of the most studied paradigms for the understanding of social interactions. Reciprocal altruism -a special type of cooperation that is taught by means of the iterated prisoner dilemma game (iPD)- has been shown to emerge in different species with different success rates. When playing iPD against a reciprocal opponent, the larger theoretical long-term reward is delivered when both players cooperate mutually. In this work, we trained rats in iPD against an opponent playing a Tit for Tat strategy, using a payoff matrix with positive and negative reinforcements, that is food and timeout respectively. We showed for the first time, that experimental rats were able to learn reciprocal altruism with a high average cooperation rate, where the most probable state was mutual cooperation (85%). Although when subjects defected, the most probable behavior was to go back to mutual cooperation. When we modified the matrix by increasing temptation rewards (T) or by increasing cooperation rewards (R), the cooperation rate decreased. In conclusion, we observe that an iPD matrix with large positive reward improves less cooperation than one with small rewards, shown that satisfying the relationship among iPD reinforcement was not enough to achieve high mutual cooperation behavior. Therefore, using positive and negative reinforcements and an appropriate contrast between rewards, rats have cognitive capacity to learn reciprocal altruism. This finding allows to infer that the learning of reciprocal altruism has early appeared in evolution.

## Introduction

Altruism is a behavior by an individual that may be to his disadvantage but benefits others individuals. At first sight, Darwin’s natural selection theory does not explain altruistic behavior. Theories have been proposed to account altruist behavior: kin selection [1], group selection and reciprocal altruism [2] among others. In the reciprocal altruism theory, the loss experienced by an individual for being altruist returns later on behalf of the reciprocal partner. Thus, in the long term, being altruist becomes the most useful strategy. In this regard, Triver’s theory of reciprocal altruism explains how natural selection favors reciprocal altruism between non-related individuals. Perhaps the most insightful example of such behavior is the one observed among vampire bats, where individuals share blood with others who have previously shared their food [3].

Since 1971, Iterated Prisoner’s Dilemma (iPD) has been a useful tool to study reciprocal altruism [4]. In the iPD, two players must choose between two possible behaviors: to cooperate or to defect. Rewards and punishments are defined in a 2x2 payoff matrix. When the game is played indefinitely, which is its iterated version, mutual cooperative behavior is favored. When played once, to defect is the best strategy [5]. However, when the game runs indefinitely, evolutionary stable strategies (ESS) emerge [6, 7] and, under certain constraints imposed to the payoff matrix, mutual cooperation appears as the best strategy whenever reciprocity is maintained (*Pareto Optimum*). Among a huge number of reciprocal strategies, tit for tat is one of the most simple ones [8]. It is based on two simple rules: to cooperate in the first trial and, in the following, to do what the other player (opponent) did in the last trial.

Among many reciprocal behaviors, reciprocity and reciprocal altruism were well documented in several species. Although cooperation is needed to succeed in both reciprocity and reciprocal altruism, the latter adds the possibility of obtaining reward by defecting an opponent. Some experiments show reciprocal altruism behavior by means of iPD paradigm in different ways, but the results were either low levels of cooperation [9] or depended on a treatment that enhanced cooperation preference (mutualism matrix) [10–12]. Direct reciprocity, which is established between two individuals, has been observed in monkeys [13–15] and in rats [16–19]. While food quality seemed to impact on cooperative behavior, a key factor to obtain reliable cooperation levels was the opponent’s behavior. In this sense, individuals tended to be more cooperative with opponents that had cooperated in the past. However, when reciprocal altruism is studied, differences between species come to light. Thus, while reciprocal altruism has been proven in monkeys, birds and rats failed to reach high levels of cooperation, even for complex combinations of rewards and punishments in the payoff matrix and treatments to induce preference [9, 10, 12, 20–23]. The reasons why some species do not learn reciprocal altruism remain obscure. A possible explanation is that animals are not able to discriminate low contrast reward contingencies. Indeed, it has been shown that rats fail to discriminate the amount of reward when the number of reward units is larger than three [24–26]. In this work, we designed an iPD setup to maximize the contrast among reinforcers. The amounts of pellets were chosen in order to minimize positive reinforcement earned in each trial and to keep rats motivated (hungry), [27]. In order to evaluate if animals developed ALLC strategy by place preference (after animals learned iPD) they were trained on reversal. We also evaluated reward maximization studying how the payoff matrix components promote or disrupt altruistic behavior.

## Materials and methods

### Subject

**Fig 1:**
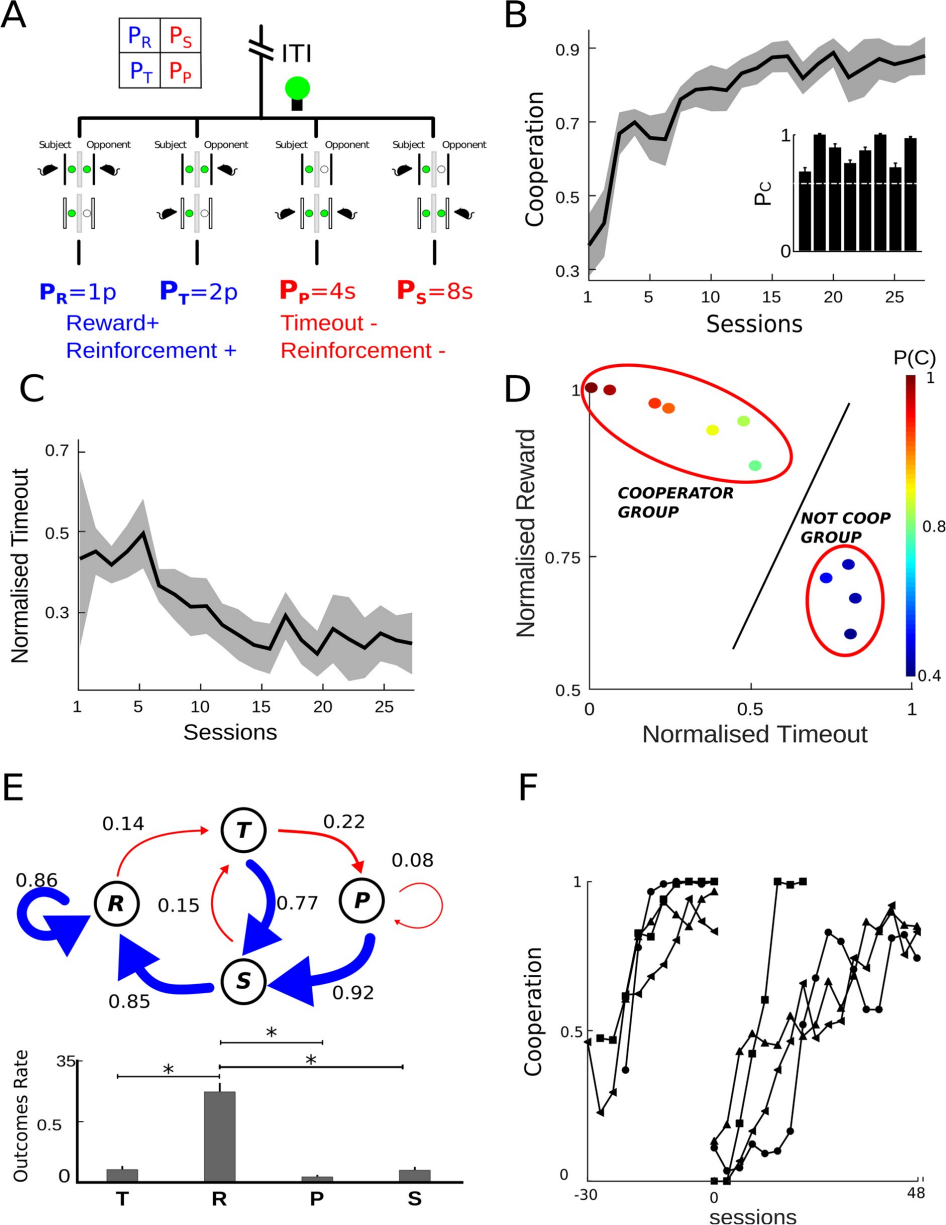
High level of cooperation in iPD. (**A**) Dual operant box diagram and the matrix with positive(blue) and negative(red) reinforcement is shown. The iPD game had four possible states: R(reward) mutual cooperation, P(punishment) mutual defection, T(temptation) in which subject defected and opponent cooperated and S(sucker) subject cooperated and opponent defected. The opponent´s light was driven in order to perform a Tit for tat strategy. (**B,C**) Time-course of cooperation and timeout rate along the last 23 games sessions. In the last 5 sessions, the mean ± sem of cooperation was 0.86 ± 0.05 and timeout was 0.23 ± 0.08. (**D**) Total reward versus timeout for all animals (color bar means cooperation mean). Each animal was compared with the regression line fit to a population with cooperation level set to 60% (black continuous line). The higher the cooperation levels, the larger the total reward and the lower the total timeout. (**E**) Markov Chain diagram shows the probabilities of transition between states (*p*(*c*|*T*_−1_) = 0.76, *p*(*c*|*R*_−1_) = 0.85, *p*(*c*|*S*_−1_) = 0.93, *p*(*c*|*P*_−1_) = 0.87). The arrow represents transitions: driven by cooperation in blue, and driven by defection in red (the arrow thickness is proportional to transition probability). Below, bars show occupancy ratio when cooperation reaches stability. Probabilities were: *p*(*R*) = 0.76, *p*(*T*) = 0.1, *p*(*P*) = 0.04, *p*(*S*) = 0.1. Asterisks denote significant differences from multiple comparisons using one-way ANOVA and Bonferroni correction. (**F**) Evolution cooperation rate before and after reversal. Graphs show a moving average with samples of 3 sessions (the mean and sem from reversal on the last five sessions was 0.87 ± 0.04).

We used thirty male Long-Evans rats (weight 300-330g and two months old) provided by the IBYME-CONICET, divided in two experiments. In the first one, eighteen rats (twelve experimental and six opponent), and in the second, twelve rats (six experimental and six opponent). Experimental subjects were housed in pairs (to allow social interaction), and opponent rats were housed individually. All rats were food restricted and maintained at 90-95% for experimental subjects, and 80-85% for opponents of free feeding body weight, all with tap water available ad libitum. The housing room was at 22°*C* ± 2°*C* and 12/12 h light/dark cycle (with lights on at 9 am). Pre-training was performed on a single standard operant chamber (MED associates Inc., USA) equipped with two stimulus light and retractable levers below the light and feeders. Also the chambers were inside an anechoic chamber with white noise (with a flat power spectral density). The iPD experiments were performed in ad hoc dual chamber equipped with levers, lights and feeders (Fig 1A). The chambers were connected by windows allowing rats to make olfactory and eye contact. The lever’s height was 80% of maximum height of the forepaws while rearing [27]. The dual chamber is shown in supplementary material (see S2 Fig). At the end of daylight, supplementary food was provided to allow rats to maintain body weight.

### Pre-experimental training

All rats had a shaping procedure to learn the response (press a lever) to get a reinforcement (pellets). To prevent animals from choosing a lever place over the other, they learned to get reward from both sides by changing the side of conditioned stimulus. The side was changed after eight trials. All rats learned to press the correct lighting lever after four sessions. Each rat was trained in 2 sessions per day, each trial began with the inter-trial interval (ITI) during 5 seconds, it was followed by the conditioning stimulus (light) for either 45 seconds or until a lever was pressed. One second before food is delivered, the feeder was lighted. In the opponent’s training, they learned to press the lever when the light was on. In the task, the side of the active lever was chosen pseudo-randomly (allowing the same side no more than four times). The opponent subject had to perform a fix ratio treatment up to FR = 5 to get rewards.

### Experiment

To study the reciprocal altruism in an iterated Prisoner’s Dilemma game (iPD), we used a payoff matrix with positive and negative reinforcements. Positive reinforcements were pellets (Bio-Serv 45 mg Dustless Precision Pellets) and negative reinforcement was timeout (a fix delay in starting a new trial). The payoff of the experimental subject was according to the matrix, and the opponent’s payoff was 1 pellet when the correct lighted lever was pressed. For the opponent, when the incorrect lever was pressed, there was no contingency and no pellet was delivered. The trial finishes after 45 seconds elapsed, or when the correct lever is pressed. The iPD game has four possible occupancy states where experimental and opponent individual behaviors can be as follows: both cooperate (mutual cooperation, R), both do not cooperate (mutual defection, P), experimental subject does not cooperate when the opponent cooperates (T), and experimental cooperates when the opponent does not cooperate (S). The amount of pellets preference was previously tested on a discrimination test, showing that rats prefer 2 pellets rather than 1 pellet (data not showed). We performed two sessions per day and each session had 30 trials. Each experimental subject was trained with the same opponent. The training was finished after five consecutive sessions with no changes in the cooperation rate. We defined cooperation (C) and defection (D) lever in the iPD box. The single iPD trial procedure was as follows: (1) ITI time, (2) then, the light (CS) was turned on, (3) after this, both rats made their responses, the light was turned off and the reinforcement was delivered according to a payoff matrix, (4) if positive reinforcement was assigned, the feeder’s light was turned on, and a second later a reward was delivered. The opponent’s Conditioned Stimulus (light) was controlled following a Tit for tat strategy. The opponent received a pellet after pressing three times the lever (FR = 3, so as to be enough time in front of the window until the experimental subject choose a lever). If negative reinforcement (timeout) was assigned, delay time started, and the opponent subject got a pellet reward. (5) After either five seconds eating time expired or timeout was completed, a new trial started. In the first experiment the payoff matrix was: 1 pellet for mutual cooperation (*P*_*R*_ = 1), 2 pellets when the experimental subject defected and the opponent cooperated (*P*_*T*_ = 2), 4 seconds of timeout for mutual defection (*P*_*P*_ = 4*seconds*), and 8 seconds of timeout when the experimental subject cooperated and the opponent defected (*P*_*S*_ = 8). At the end of these experiments, the four rats with the best performance in cooperation were trained in a reversion treatment (see Fig 1F). When rats were trained on reversal, the sides of C and D lever were interchanged in subject and opponent chambers. In that sense, if animals developed a place-preference behavior, they will not learn the new side in order to maximize reward. In the second experiment we used six naive experimental rats on a different payoff matrix with greater temptation (*P*_*R*_ = 1, *P*_*T*_ = 3, *P*_*P*_ = 4, *P*_*S*_ = 8). After training, we divided rats in two groups, depending on cooperation levels. The first group (Treat 2A) with high cooperation rate was trained with the payoff matrix (*P*_*R*_ = 1, *P*_*T*_ = 5, *P*_*P*_ = 4, *P*_*S*_ = 8) with greater temptation for T state (Treat 3A). The other group (with low cooperation rate, Treat 2B) was trained with the matrix (*P*_*R*_ = 2, *P*_*T*_ = 3, *P*_*P*_ = 4, *P*_*S*_ = 8, Treat 3B) that enhances cooperative behavior (in comparison with (*P*_*R*_ = 1, *P*_*T*_ = 3, *P*_*P*_ = 4, *P*_*S*_ = 8), but with low contrast between positive rewards (see Table 1). All experimental procedures were approved by the ethics committee of the IByME-CONICET and were conducted according to the NIH Guide for Care and Use of Laboratory Animals.2.1 Subjects and Housing.

**Table 1:** Data summary. Treatment 1: testing of high cooperation and reversion. Treatment 2 and 3: effect in cooperation by change of pay-off matrix. The matrix changed over the group with same word (A or B).

Treat	Groups	Cooperation	Probability State				Transition Probabilities			
			p(T)	p(R)	p(S)	p(p)	*p*(*c*|*T*_1_)	*p*(*c*|*R*_1_)	*p*(*c*|*S*_1_)	*p*(*c*|*P*_1_)
1	coop	0.86 ± 0.05	0.10	0.76	0.1	0.04	0.76	0.85	0.93	0.87
	no coop	0.36 ± 0.03	0.44	0.38	0.32	0.32	0.25	0.19	0.33	0.23
	reversal	0.87 ± 0.04	0.11	0.77	0.02	0.10	0.86	0.89	1.00	0.82
2	A	0.87 ± 0.04	0.09	0.80	0.03	0.08	0.65	0.90	0.87	0.94
	B	0.64 ± 0.13	0.23	0.34	0.21	0.22	0.47	0.55	0.56	0.65
3	A	0.61 ± 0.10	0.18	0.44	0.21	0.17	0.45	0.64	0.62	0.78
	B	0.71 ± 0.04	0.20	0.51	0.09	0.20	0.62	0.66	0.67	0.66

#### Statistic

All statistical analyses were performed using statistics library from open source software Octave and MATLAB. We pooled the data from the last five sessions where cooperation rate was stable (to calculate cooperation rate we counted the number of times a rat chose the cooperation lever per session). We compared individual’s means of cooperation along treatment using a two-sided Wilcoxon rank sum test. To test whether the probability of cooperation after each outcome (T, R, P or S) was different from chance (0.5), we performed a Chi-square goodness of fit test with Bonferroni corrected value of 0.05/n. To compare mean rate of the different outcomes for each game, we performed an ANOVA two tails test. When significant *α* = 0.05, multiple post-hoc pairwise comparative tests were performed with Bonferroni corrected value of *α* = 0.0125. The individual’s decision rules can be described by the components of transition vectors and Markov Chain diagram. The transition vector was made up of probabilities of cooperation when the previous trials resulted in state *p*(*c*|*R*_−1_), T(temptation) *p*(*c*|*T*_−1_), S(sucker) *p*(*c*|*S*_−1_) or P(punishment), *p*(*c*|*P*_−1_) respectively. If every component of this vector is 0.5, the agent’s decision rule is random mode. Markov Chain diagram show the graphic representation of the complete decision making rule for each rat.

## Results

We trained twelve rats in iPD against an opponent that plays Tit for tat strategy. Tit for tat is based on two simple rules: to cooperate in the first trial and, in the following, to do what the other player (opponent) did in the last trial. Fig 1A shows a schema of the different choices a subject can do in each trial. Thus, when the subject cooperates, it receives one pellet (*P*_*R*_) or eight seconds timeout (*P*_*S*_) depending on whether the opponent choice was to cooperate or to defect. On the other hand, when the subject defects, it receives 2 pellets (*P*_*T*_) or four seconds timeout (*P*_*P*_), according to whether the opponent choice was to cooperate or to defect respectively. The criteria for cooperation was an established preference for pressing C lever (cooperation) over D lever (defection) in more than 60% of the trials for five or more consecutive sessions. Eight out of twelve animals learned to cooperate (cooperation rate 0.86 ± 0.05, mean ± s.e.m), reaching criteria in 30 ± 4 sessions (mean ± s.e.m). In Fig 1B, we show the mean cooperation levels for those animals during the last twenty three sessions before reaching criteria. The inset in Fig 1B shows the mean cooperation level for each animal during the last five training sessions. As a consequence of the increase in cooperation levels, the average total timeout per session decreased as training progressed (0.23 ± 0.08, mean ± sem, see Fig 1C).

Due to the fact that different sequences of lever pressing can give the same amount of reward and/or timeout independently of the cooperation level, we analyzed the relationship between total reward and timeout for each animal in comparison to a simulated population. A regression line was fit to a population of 100,000 simulated individuals with cooperation level set to 60%, (see Fig 1D). Each simulated individual had one different strategy and each one was a combination of thirty C and D choices (session length). An individual that plays an iPD game with 60% of its choices in C will be near to the line, regardless of its strategies. As it can be seen in the figure, for the cooperator group when the cooperation level increases, the larger are the total reward, and the lower the total timeout. For the non cooperator group placed in the opposite side of the figure, it can be seen that both cooperation and reward were low and timeout was high. The regression line at 60% of cooperation separates both groups (marked with a red circle in the Fig 1D). This shows that no behavior with low level of cooperation (subgroup in blue range) can obtain both high level of reward and small amount of timeout as in the cooperative group. The average strategies of both group can be represented by Markov model diagram. We built one Markov model for the group of cooperative animals (see Fig 1E) averaging occupancy state rate and transition probabilities in the group. In the iPD there are four possible occupancy states where experimental and opponent individual behaviors can be as follows: R (both cooperate or mutual cooperation), P (both do not cooperate or mutual defection), T (experimental subject does not cooperate when the opponent cooperates), and S (experimental cooperates when the opponent does not cooperate). The cooperative group showed that the permanency in R state was high and, whenever the animal defects (states T and P), it returns to cooperate immediately. Indeed all conditional probabilities to cooperate given a previous outcome were near 1. Besides, the rate of R state was the highest and other states near zero. The probability of R state was significantly different to other states (*p* = < 1*e*^−8^, ANOVA two-way test, n = 8). On the contrary, in the group of non-cooperative animals, any states were significantly different to the other *p* > 0.05, F = 0.353, ANOVA two-way test, n = 4) and the probability to cooperate given a previous states did not evidence preference for any defined strategy (see Table 1 conditional probability to cooperate). For the group of non-cooperative animals Markov model (see S1 Fig, supplementary materials).

To discard the fact that animals had a preference for one of the levers and, in consequence, their behavior biased independently of the training paradigm, we selected the best four cooperators and applied a reversal procedure immediately after cooperation was reached. All animals learned to cooperate after reversal (cooperation rate, 0.87 ± 0.04, mean ± sem), (see Fig 1F).

We then asked how the ratio in the amount of positive reinforcement of R and T states affects cooperation learning and maintenance. We defined a contrast index CI that measures the relationship between the amount of reward in R and T as follows:

$$\begin{array}{c} CI=\frac{P_{T}-P_{R}}{P_{T}+P_{R}} \end{array}$$

**Fig 2:**
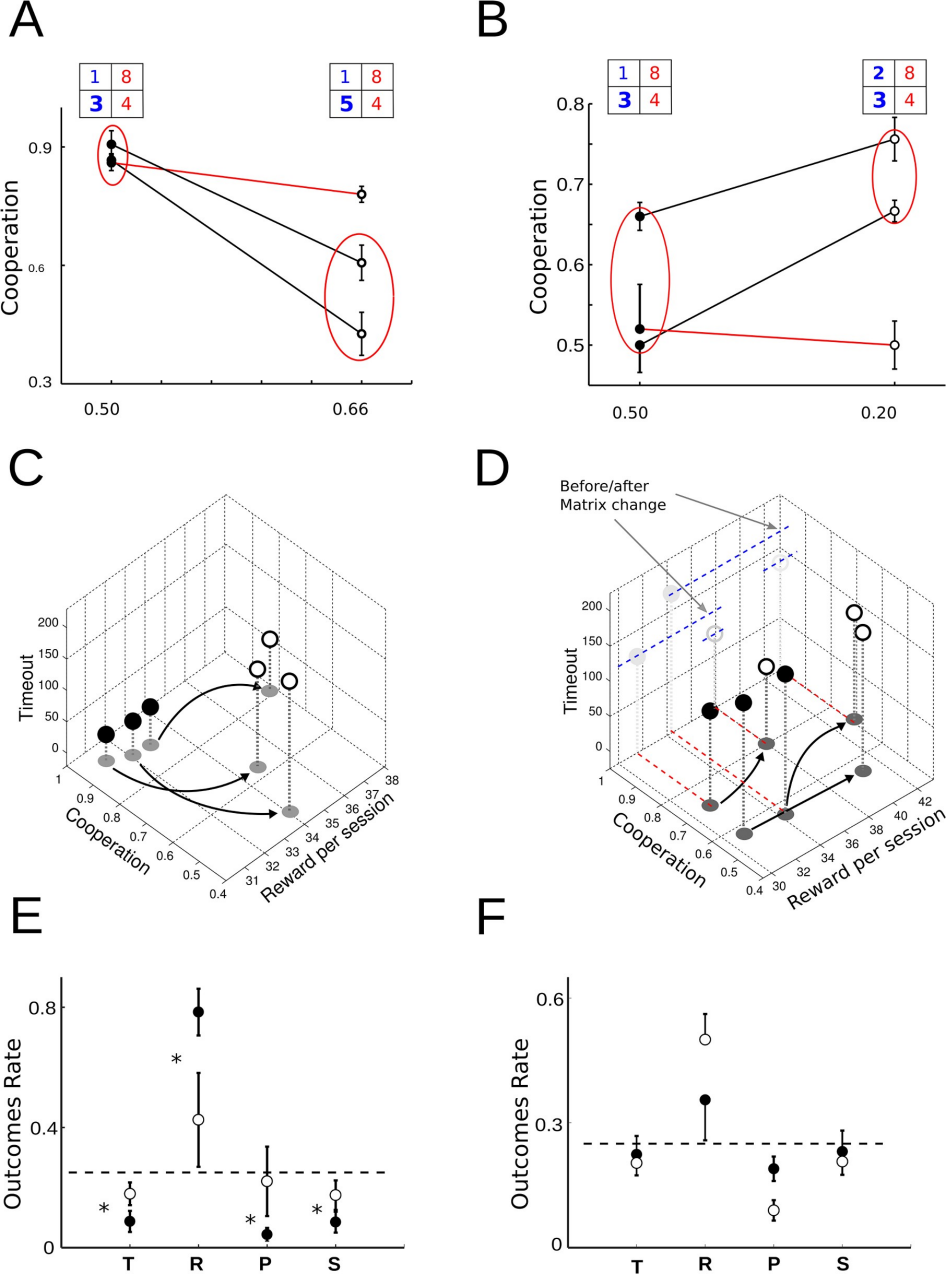
Effect of changes in the amount of positive reinforcement of R and T. (**A**) The rats were pre-trained by pay-off matrix [*P*_*R*_ = 1, *P*_*T*_ = 3, *P*_*P*_ = 4, *P*_*S*_ = 8 and contrast $CI=\frac{1}{2}$] (filled dots) and the cooperation was strongly affected by change of temptation payoff, decreasing when T payoff increased and matrix with changed to [*R* = 1, *T* = 5, *P* = 4, *S* = 8 and contrast $CI=\frac{2}{3}$] (open circles). There was a significant difference (red circle) in two animals with *p* < 9.8*e*^−06^ (wilcoxon rank-sum test) and the other did not modify her behavior in spite of matrix change. (**B**) The cooperation enhanced when the matrix changed to [*R* = 2, *T* = 3, *P* = 4, *S* = 8 and $CI=\frac{1}{5}$] (open circles) and the difference was statistically different (*p* < 0.0062) in two of three subjects, because one had no significant difference after matrix change, *p* > 0.05(*cooperation*: 0.7063). (**C**) The 3D plots related cooperation, reward and timeout. In the group of cooperative animals (filled dots), the change in T (3 pellets to 5 pellets) increased both timeout and reward in order to decrease cooperation (open circles). The comparison between cooperation mean of both groups was significantly different, *p* < 0.05. (**D**) In the group of non-cooperative animals (filled dots), they learned to cooperate (open circles) by receiving more reward without significant changes in total timeout. The cooperation was significantly different, *p* > 0.05. (**E**,**F**) The mean of occupancy state rate graph (last five sessions) from cooperative (left) and non-cooperative (right) groups (Mean ± sem). Asterisks denote significant difference, after matrix changed, among T, R, P or S state occupancy and dash line indicates the level of equal rate in each state (that corresponds to a strategy with strongly random component). Before changes (filled dots) and after changes (open circles).

Thus, in the experiment shown in Fig 1, the CI was $\frac{1}{3}$ which is the maximum contrast level constrained to a payoff matrix that favors cooperation, that is, 2*P*_*R*_ > *P*_*T*_ + *P*_*S*_, assuming that S becomes a negative stimulus induced by timeout. We trained six animals with a payoff matrix (*P*_*R*_ = 1, *P*_*T*_ = 3, *P*_*P*_ = 4, *P*_*S*_ = 8) and found that three animals learned to cooperate (0.88 ± 0.01, mean ± sem, see Fig 2A), while others did not (0.64 ± 0.13, mean ± sem, see Fig 2B. The last group was non cooperator, since both their conditional probabilities to cooperate and occupancy R state ratios were near chance. For details see Table 1. Then we changed the amount of reward in order to increase/decrease CI in the cooperative/non-cooperative groups. As it can be seen, a high value of $CI=\frac{2}{3}$, related to a pay-off matrix (*P*_*R*_ = 1, *P*_*T*_ = 5, *P*_*P*_ = 4, *P*_*S*_ = 8), disrupts cooperation in cooperative group, Fig 2A. The cooperation was 0.604 ± 0.102, mean ± sem whereas before 0.88 ± 0.01). When a lower value of $CI=\frac{1}{5}$ was applied for non cooperator group and the matrix (*P*_*R*_ = 2, *P*_*T*_ = 3, *P*_*P*_ = 4, *P*_*S*_ = 8) empowers the cooperation in two out of three animals, cooperation rate 0.711 ± 0.04, mean ± sem, whereas before 0.64 ± 0.13 (see Table 1).

We analyzed how these changes in strategies impact on the amount of received reward and timeout penalties. In the group of cooperative animals, the change in T (3 pellets to 5 pellets) increased both timeout and only a bit reward, as expected when states T, P and S become more probable. The occupancy states ratio before and after matrix change had significant differences among all states, *p* < 0.05 (wilcoxon ranksum test), (see Fig 2C and 2E). It is worth noting however that the amount of received reward is not the maximum allowed, which would be delivered in the case of an animal that alternates from state T to S indefinitely. On the other hand, when we applied a matrix with a lower contrast $CI=\frac{1}{5}$ to the group of non-cooperative animals, they enhance significantly their cooperation level, receiving more reward without significant changes in total timeout, (see Fig 2D). In Fig 2F, we show the state occupancy probabilities for this group before and after the change in the payoff matrix. It can be seen that the occupancy state ratio of R had significantly increased after the change in the payoff matrix. It can be observed a significant difference in R and P states, (*p*_*R*_ < 0.008 and *p*_*P*_ < 0.048, wilcoxon rank-sum test). We showed that when the contrast index increased using a matrix to favor cooperation the animals learned to cooperate, but when the index increased and the matrix favor defection the animals stopped cooperating.

**Fig 3:**
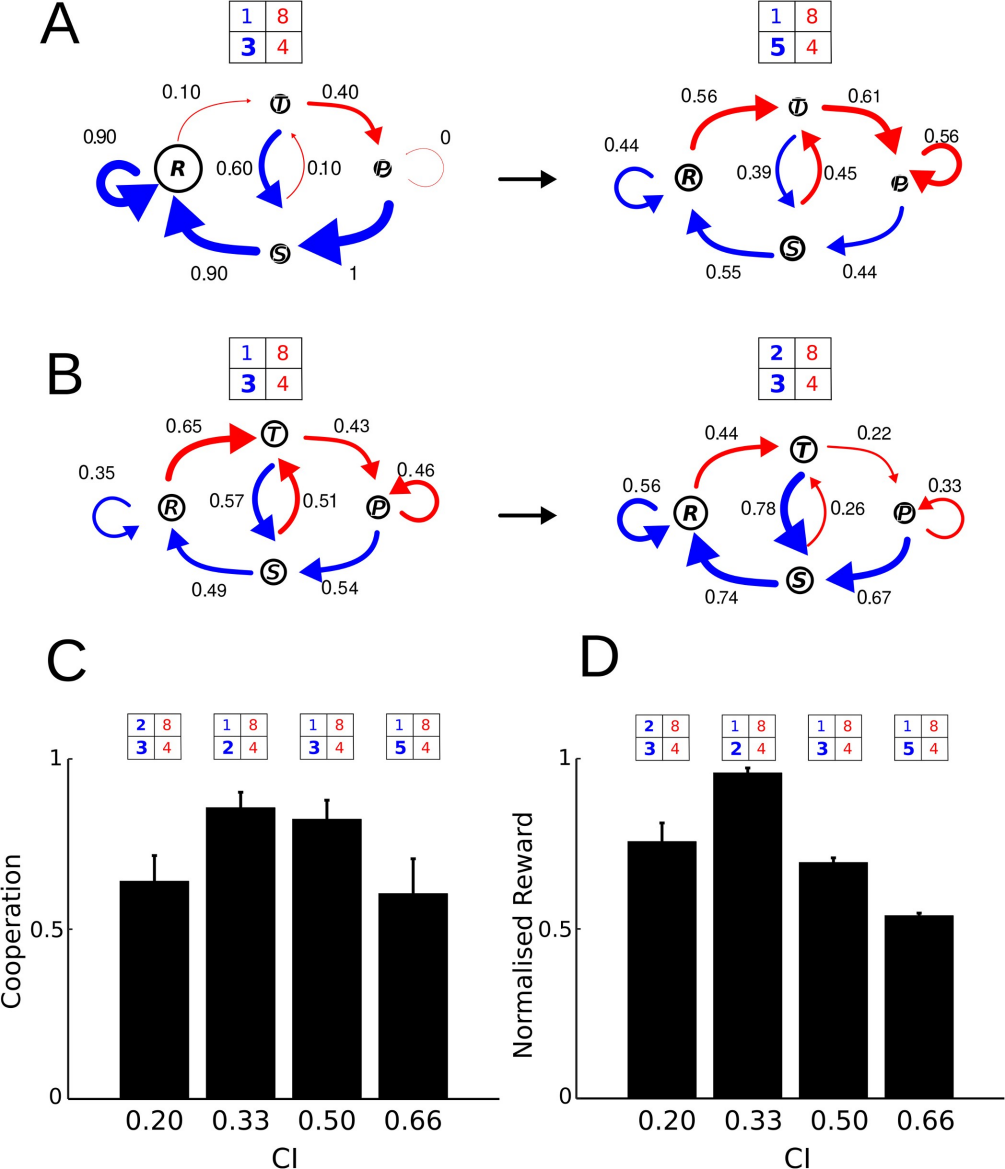
Markov chain diagrams and contrast index. Markov chain diagrams are shown (the size of circle means of occupancy state rate and the arrow’s width are proportional to the probability of cooperate given (**A**) occupancy state and transition probabilities for an animal that disrupted its cooperative behavior when contrast index $CI=\frac{1}{2}$ was changed to $CI=\frac{2}{3}$ and pay-off matrix was changed [*P*_*T*_, *P*_*R*_, *P*_*P*_, *P*_*S*_] = [3*p*, 1*p*, 4*s*, 8*s*] to [5*p*, 1*p*, 4*s*, 8*s*] (p = pellet and s = seconds). The thickness of blue arrows (conditional probabilities of cooperation) become thinner after change (for values see Table 1). (**B**) The opposite situation can be seen, non-cooperative animal becomes more cooperative when $CI=\frac{1}{2}$ was decreased to $CI=\frac{1}{5}$ in a matrix that favors cooperation. The blue arrows become thicker after change (for values see Table 1). (**C**, **D**) shows cooperation and timeout levels as a function of CI. Here, it can be seen that both variables follow an inverted U profile in correlation with the contrast index increase and if the payoff matrix favors or not the cooperation behavior.

From the results shown in Figs 1 and 2, it is reasonable to ask whether a fine tuning in contrasted reward encourages cooperative behavior. We have shown that eight out of twelve animalas (66%) acquired a cooperative behavior when CI was $\frac{1}{3}$, while three out of six (50%) succeeded when CI was $\frac{1}{2}$, as expected when temptation payoff increases. In the same line of reasoning, animals that learned cooperation under $\text{CI}=\frac{1}{2}$ disrupted their cooperative behavior when CI was increased to $\frac{2}{3}$, while those that had not learned acquired a cooperative behavior when CI was decreased to $\frac{1}{5}$. Fig 3A exemplifies the occupancy and transition probabilities for an animal that disrupted its cooperative behavior when $CI=\frac{1}{2}$ was changed to $CI=\frac{2}{3}$. The opposite can be seen in the example of Fig 3B. A non-cooperative animal under a $CI=\frac{1}{2}$ became cooperative when CI was decreased to $\frac{1}{5}$. Fig 3C and 3D show cooperation levels and normalized rewards. A normalized reward was calculated as quotient between the total reward obtained in a session, and the maximum reward achieved using the best strategy. If the opponent subject plays a Tit for tat strategy, the best strategy will depend on the pay-off matrix values. In this way, if the matrix favors cooperation, ALLC will be the best one. In contrast, when the payoff matrix favors no cooperation, alternate between C and D will be the best strategy. It can be seen that both variables follow an inverted U profile as a function of contrast index CI, as expected when a delicate balance between rewards at R and T is mandatory.

## Discussion and conclusion

In this work, we study the contrasted role between reinforcements in the learning of reciprocal altruism learning in rats. Traditionally, reciprocal altruism is achieved by playing the iterated prisoner’s dilemma game (iPD) when an experimental subject is confronted to a reciprocal opponent. The payoff matrix used has positive and negative reinforcements with high contrasted between positive and negative pairs and also uses discriminating amount of reinforcements [25, 26]. In our experiment, pellets were used as positive reinforcements, and timeout as negative reinforcement. In this way, the positive and negative reinforcements acted as strengtheners of mutual cooperation behavior likelihood [28]. Our results show for the first time high levels of cooperation (86,11%) and mutual cooperation (76,32%) in iPD, (see Fig 1B). Previous published works have taught reciprocity using iPD game, showing that animals prefer short-term benefits or only improve a poor level of cooperation [4, 9, 20, 29, 30]. In other works, authors employed a special treatment to enhance cooperation preference [10, 23, 31, 32]. A possible explanation is that using standard matrices (for example: *P*_*T*_ = 6, *P*_*R*_ = 4, *P*_*P*_ = 1, *P*_*S*_ = 0), animals were not able to discriminate between the amount of reinforcement obtained in the long-term in comparison to short-term [24]. For example, if a rat played four sessions [C C C C] he would get 16 pellets, and if played [C D D D] he would get 12 pellets. In our experiment, rats using the same choices earn 4 pellets and no timeout in the first case, and 3 pellets plus a 16 seconds timeout in the second case.

A dynamic system can be represented with Markov diagrams and its associated state transition vector. In this case, each state (T, R, P, S, see Results section) will have two associated conditional probabilities: to cooperate or not to cooperate given state. In an IPD game with an opponent using a Tit for tat strategy, a rational player should maximize the positive reinforcement and cancel the negative reinforcement. In this way, while the opponent performed a reciprocal behavior, the player follows an ALLC strategy with conditional cooperation probability near 1, independent of previous states (T, R, P o S). In a pay-off matrix with addable value (as for an example (*P*_*T*_ = 6, *P*_*R*_ = 4, *P*_*P*_ = 1, *P*_*S*_ = 0), it is possible to calculate the cooperative strategy through mathematical analysis [33, 34], but in our experiment positive and negative reinforcers have different units (pellets and time respectively). Due to this reason, we did a single analysis using the Markov chain diagram. In the first experiment, we found that animals adopted two well defined strategies. On one hand, a group of 8 animals proved to have learned a cooperative strategy while other 4 animals responded at random (see S1B Fig, Supporting information). The strategy of the first group, (see Fig 1E), show that conditional probabilities to cooperation given previous state T, R, P or S were near 1 (0.760, 0.845, 0.929 and 0.870, respectively) and in this fashion after defected they immediately return to the mutual cooperation state, R. In various works, results were presented with Markov diagrams and its associated transition vector [10, 11, 23, 32] and showed that conditional probabilities of cooperation were not high when facing a reciprocal opponent. In this protocol, with the matrix (*P*_*T*_ = 2, *P*_*R*_ = 1, *P*_*P*_ = 4*s*, *P*_*S*_ = 8*s*), there are two theoretical strategies that maximize appetite reinforcement: one is ALLC strategy and the other an alternating between cooperation (C) and defection (D) strategy. The latter, also maximizes positive reinforcement when alternating between cooperation and defection options, but it also increases negative reinforcement (timeout). In this case, ALLC strategy is the only one that maximizes positive reinforcement and minimizes the negative one (Pareto Optimum). Since negative reinforcement is timeout, ALLC strategy gives more food per unit of time. In this case, the role of the negative reinforcement appears.

In order to evaluate if animals developed ALLC strategy by place preference (after animals learned iPD) or by reward maximization, they were trained on reversal, (see Fig 1F), and we observed that animals relearn reciprocal altruism when they are exposed to a new lever’s contingency.

Finally, after animals adopted a strategy, we evaluated if a change in the payoff matrix could modify their behavior. Therefore, we studied the effect of modifying positive reinforcements (see Fig 2A and 2B). Animals were pre-trained with a payoff matrix where alternating between C and D strategy gives more positive reinforcements than with an ALLC strategy, keeping the same negative reinforcement as in the first experiment. We observed that only half of the animals learned to cooperate although all of them obtained the same mean amount reward (pellet) (see Fig 2C and 2D). The cooperative group was trained with a matrix where the pay-off T was increased (Fig 2A), then we observed that cooperative behavior decreased. Animals reduced frequency of R state and increased frequency of P state, proving that they preferred a small-immediate option instead of a large-delayed option. This behavior is similar to the one observed in birds ([30]). In the second group, we applied a matrix that keeps the proportions of reinforcements in T and R similar to the most common matrix (*P*_*T*_ = 3*p*, *P*_*R*_ = 2*p* equal proportion to *P*_*T*_ = 6*p*, *P*_*R*_ = 4). It was observed that animals modified their behavior and became more cooperative (Fig 2B). These results show that rats that learned to cooperate with an appropriate matrix stop cooperating when a temptation payoff (T) is sufficiently increased (matrix with high contrast index). However, if non-cooperative animals are trained with a matrix that favors cooperation (matrix with low contrast index), they become cooperators. In the latter case, the achieved cooperation level was comparable to results shared in diverse bibliography. We observe that if an iPD matrix uses large positive reward, it improves less cooperation than one with small rewards, shown that satisfying the relationship among iPD reinforcement was not enough to achieve high mutual cooperation behavior. The reciprocal altruist behavior in humans, monkeys and elephants has been studied in laboratories showing high levels of cooperation [13, 15, 35–37], however in rats and birds those levels of cooperation were much lower. Our results show that by using positive and negative reinforcements and an appropriate contrast between rewards, rats have cognitive capacity to learn reciprocal altruism. This finding allows to deduce learning of reciprocal altruism appeared early in evolution.

## Supporting information

S1 Fig Non-cooperative rats. (**A**) Time-course of cooperation rate along the last 23 game sessions. In the last 5 sessions, the mean ± sem of cooperation was 0.36 ± 0.03. (**B**) Markov Chain diagram shows the probabilities of transition between states (*p*(*c*|*T*_−1_) = 0.44, *p*(*c*|*R*_−1_) = 0.38, *p*(*c*|*S*_−1_) = 0.32, *p*(*c*|*P*_−1_) = 0.32). The arrow represents transitions: driven by cooperation in blue, and driven by defection in red (the arrow thickness is proportional to transition probability). The size of circles is proportional to the state occupancy ratio. Below, bars show the occupancy ratio (*T* = 0.25, *R* = 0.19, *P* = 0.33, *S* = 0.23 and *p* > 0.05, F = 0.353, ANOVA two-way test, n = 4) and transition probabilities (*p*(*c*|*T*_−1_) = 0.43, *p*(*c*|*R*_−1_) = 0.38, *p*(*c*|*S*_−1_) = 0.32, *p*(*c*|*P*_−1_) = 0.31) did not evidence preference for any defined strategy. Asterisks denote significant differences from multiple comparisons using one-way ANOVA and Bonferroni correction.
(EPS)

S2 Fig Scheme of dual operand conditioning chamber. Two operant boxes are placed, one in front of the other in such a way that transparent windows were aligned. In the front panel of each box, there are two light stimulus (green = on / black = off) on the top, two levers in the middle and two windows (red shadow) in down. In the subject box both lights are turned on at the same time when the trial starts, and in the opponent box only a light is on. The opponent was trained to choose the side where the light is on, but the subject has to learn which side maximizes reward.
(EPS)

S3 Fig Abbreviations list. (EPS)

S4 Fig Dataset of all treatments. (PDF)
