# Supplementary material for: High mutual cooperation rates in rats learning reciprocal altruism: The role of payoff matrix
Source: PLoS One. 2019 Jan 2;14(1):e0204837. doi: 10.1371/journal.pone.0204837 (PMC6314613; doi:10.1371/journal.pone.0204837)
Supplement: S4 Fig — (PDF) [file pone.0204837.s004.pdf]

TREATMENT 1

		OPPONENT	
Matrix		C	D
	C	1pellet	8second
SUBJECT	D	2pellet	4second

REWARD: rate of maximum food in the game

Session	Rat 1	Rat 2	Rat 3	Rat 4	Rat 5	Rat 6	Rat 7	Rat 8	Rat 9	Rat 10	Rat 11	Rat 12
1	0.97	0.86	1.00	0.93	0.80	0.87	1.00	0.93	0.86	1.00	0.97	1.00
2	0.93	0.67	1.00	0.77	0.70	0.73	0.93	0.93	0.97	1.00	0.90	1.00
3	0.93	0.83	1.00	0.67	0.76	0.73	1.00	0.97	0.90	1.00	0.90	1.00
4	0.93	0.77	1.00	0.62	0.63	0.63	0.93	1.00	1.00	1.00	1.00	1.00
5	0.80	0.90	1.00	1.03	0.57	0.63	0.93	0.80	1.00	1.00	0.93	1.00
6	0.73	0.70	1.00	0.83	0.27	0.73	1.00	0.93	1.00	1.00	0.97	1.00
7	0.90	0.67	1.00	0.77	0.60	0.83	1.00	0.97	1.00	1.00	0.97	1.00
8	0.80	0.67	1.00	0.67	0.72	0.63	1.00	0.93	1.00	1.00	1.00	1.00
9	0.93	0.60	1.00	0.53	0.60	0.52	0.97	0.97	0.97	1.00	0.93	0.97
10	0.90	0.70	1.00	0.33	0.67	0.53	1.00	0.93	1.00	1.00	0.93	1.00

TIMEOUT accumulated in a session (Punish + ITI)

Session	Rat 1	Rat 2	Rat 3	Rat 4	Rat 5	Rat 6	Rat 7	Rat 8	Rat 9	Rat 10	Rat 11	Rat 12
1	210	237	150	226	254	222	166	190	200	150	186	150
2	206	234	150	238	238	258	198	202	194	158	210	174
3	190	241	150	230	241	230	174	198	196	150	210	158
4	206	230	150	245	250	250	206	178	166	150	166	166
5	214	246	150	190	262	242	198	210	158	150	226	150
6	254	254	150	238	262	258	150	194	158	150	222	150
7	206	258	150	234	254	258	166	178	174	150	230	150
8	222	250	158	250	229	254	174	216	174	150	191	158
9	206	250	150	266	238	258	162	186	186	150	214	171
10	202	262	150	262	242	258	150	206	190	150	218	150

OCCUPANCY STATE rate per session of R (mutual cooperation)

Sessions	1	2	3	4	5	6	7	8	9	10
Rat 1	0.50	0.53	0.67	0.53	0.47	0.13	0.50	0.40	0.53	0.57
Rat 2	0.23	0.27	0.20	0.30	0.17	0.10	0.07	0.13	0.13	0.03
Rat 3	1.00	1.00	1.00	1.00	1.00	1.00	1.00	0.93	1.00	1.00
Rat 4	0.33	0.23	0.33	0.21	0.63	0.23	0.30	0.13	0.00	0.07
Rat 5	0.13	0.23	0.21	0.17	0.03	0.07	0.13	0.31	0.27	0.20
Rat 6	0.40	0.07	0.33	0.17	0.23	0.07	0.10	0.10	0.10	0.07
Rat 7	0.87	0.60	0.80	0.53	0.60	1.00	0.87	0.80	0.90	1.00
Rat 8	0.67	0.53	0.57	0.73	0.47	0.60	0.77	0.45	0.70	0.53
Rat 9	0.57	0.63	0.62	0.87	0.93	0.93	0.80	0.80	0.70	0.67
Rat 10	0.93	1.00	1.00	1.00	1.00	1.00	1.00	1.00	1.00	1.00
Rat 11	0.70	0.50	0.50	0.87	0.33	0.37	0.30	0.66	0.47	0.40
Rat 12	1.00	0.80	0.93	0.87	1.00	1.00	1.00	0.93	0.83	1.00

OCCUPANCY STATE rate per session of S (sucker state)

Sessions	1	2	3	4	5	6	7	8	9	10
Rat 1	0.23	0.20	0.13	0.20	0.17	0.30	0.17	0.20	0.20	0.17
Rat 2	0.27	0.17	0.27	0.20	0.33	0.27	0.27	0.23	0.20	0.30
Rat 3	0.00	0.00	0.00	0.00	0.00	0.00	0.00	0.00	0.00	0.03
Rat 4	0.27	0.23	0.17	0.21	0.17	0.27	0.23	0.23	0.23	0.13
Rat 5	0.33	0.20	0.24	0.23	0.23	0.10	0.23	0.17	0.17	0.20
Rat 6	0.23	0.30	0.20	0.23	0.20	0.30	0.37	0.23	0.21	0.20
Rat 7	0.03	0.07	0.17	0.10	0.20	0.17	0.00	0.07	0.10	0.03
Rat 8	0.13	0.17	0.17	0.10	0.13	0.13	0.10	0.24	0.13	0.20
Rat 9	0.13	0.17	0.14	0.07	0.03	0.03	0.10	0.10	0.13	0.17
Rat 10	0.17	0.17	0.15	0.10	0.07	0.03	0.10	0.00	0.00	0.03
Rat 11	0.13	0.20	0.20	0.07	0.27	0.27	0.30	0.17	0.23	0.23
Rat 12	0.13	0.00	0.10	0.03	0.07	0.00	0.00	0.00	0.03	0.07

OCCUPANCY STATE rate per session of T (temptation state)

Sessions	1	2	3	4	5	6	7	8	9	10
Rat 1	0.23	0.20	0.13	0.20	0.17	0.30	0.20	0.20	0.20	0.17
Rat 2	0.30	0.20	0.30	0.23	0.37	0.30	0.30	0.27	0.23	0.33
Rat 3	0.00	0.00	0.00	0.00	0.00	0.00	0.00	0.00	0.00	0.03
Rat 4	0.30	0.27	0.17	0.21	0.20	0.30	0.23	0.27	0.27	0.13
Rat 5	0.33	0.23	0.28	0.23	0.27	0.10	0.23	0.21	0.17	0.23
Rat 6	0.23	0.33	0.20	0.23	0.20	0.33	0.37	0.27	0.21	0.23
Rat 7	0.03	0.07	0.17	0.10	0.20	0.17	0.00	0.07	0.10	0.03
Rat 8	0.13	0.20	0.20	0.13	0.17	0.17	0.10	0.24	0.13	0.20
Rat 9	0.13	0.17	0.14	0.07	0.03	0.03	0.10	0.10	0.13	0.17
Rat 10	0.10	0.07	0.03	0.10	0.00	0.00	0.03	0.00	0.00	0.00
Rat 11	0.13	0.20	0.20	0.07	0.30	0.30	0.33	0.17	0.23	0.27
Rat 12	0.13	0.00	0.10	0.03	0.07	0.00	0.00	0.00	0.03	0.07

OCCUPANCY STATE rate per session of P (Punishment state)

Sessions	1	2	3	4	5	6	7	8	9	10
Rat 1	0.23	0.20	0.13	0.20	0.17	0.30	0.20	0.20	0.20	0.17
Rat 2	0.30	0.20	0.30	0.23	0.37	0.30	0.30	0.27	0.23	0.33
Rat 3	0.00	0.00	0.00	0.00	0.00	0.00	0.00	0.03	0.00	0.00
Rat 4	0.30	0.27	0.17	0.21	0.20	0.30	0.23	0.27	0.27	0.13
Rat 5	0.33	0.23	0.28	0.23	0.27	0.10	0.23	0.21	0.17	0.23
Rat 6	0.23	0.33	0.20	0.23	0.20	0.33	0.37	0.27	0.21	0.23
Rat 7	0.03	0.07	0.17	0.10	0.20	0.17	0.00	0.07	0.10	0.03
Rat 8	0.07	0.10	0.07	0.03	0.23	0.10	0.03	0.07	0.03	0.07
Rat 9	0.13	0.17	0.14	0.07	0.03	0.03	0.10	0.10	0.13	0.17
Rat 10	0.15	0.10	0.07	0.03	0.10	0.00	0.00	0.03	0.00	0.00
Rat 11	0.13	0.20	0.20	0.07	0.30	0.30	0.33	0.17	0.23	0.27
Rat 12	0.13	0.00	0.10	0.03	0.07	0.00	0.00	0.00	0.03	0.07

COOPERATION

Number of COOPERATION option in a session

Sessions	Rat 1	Rat 2	Rat 3	Rat 4	Rat 5	Rat 6	Rat 7	Rat 8	Rat 9	Rat 10	Rat 11	Rat 12
1	22	15	30	18	14	19	28	24	21	30	25	29
2	22	13	29	14	13	11	23	21	24	29	21	27
3	24	14	30	15	13	16	27	22	22	30	21	29
4	22	15	30	12	12	12	22	25	28	30	28	28
5	19	15	29	24	8	13	23	18	29	29	18	30
6	18	11	30	15	5	11	30	22	29	29	19	29
7	20	10	30	16	11	14	28	26	27	30	18	30
8	18	11	28	11	14	10	27	20	27	29	24	29
9	22	10	29	7	13	9	28	25	25	30	21	26
10	22	10	30	6	12	8	30	26	26	29	24	30

Markov Chain Matrix for each rat

Rat 1	T	R	P	S
T	0	0	0.47	0.53
R	0.31	0.69	0	0
P	0	0	0.36	0.64
S	0.33	0.67	0	0

Rat 7	T	R	P	S
T	0	0	0.22	0.78
R	0.09	0.91	0	0
P	0	0	0.2	0.8
S	0.12	0.88	0	0

Rat 2	T	R	P	S
T	0	0	0.53	0.47
R	0.59	0.41	0	0
P	0	0	0.55	0.45
S	0.67	0.33	0	0

Rat 8	T	R	P	S
T	0	0	0.36	0.64
R	0.25	0.75	0	0
P	0	0	0.32	0.68
S	0.14	0.86	0	0

Rat 3	T	R	P	S
T	0	0	0	1
R	0	1	0	0
P	0	0	0	0
S	0	1	0	0

Rat 9	T	R	P	S
T	0	0	0.22	0.78
R	0.15	0.85	0	0
P	0	0	0.22	0.78
S	0	1	0	0

Rat 4	T	R	P	S
T	0	0	0.5	0.5
R	0.38	0.63	0	0
P	0	0	0.65	0.35
S	0.6	0.4	0	0

Rat 10	T	R	P	S
T	0	0	0	0
R	0	1	0	0
P	0	0	0	0
S	0	0	0	0

Rat 5	T	R	P	S
T	0	0	0.65	0.35
R	0.57	0.43	0	0
P	0	0	0.65	0.35
S	0.54	0.46	0	0

Rat 11	T	R	P	S
T	0	0	0.26	0.74
R	0.31	0.69	0	0
P	0	0	0.16	0.84
S	0.3	0.7	0	0

Rat 6	T	R	P	S
T	0	0	0.51	0.49
R	0.63	0.37	0	0
P	0	0	0.62	0.38
S	0.61	0.39	0	0

Rat 12	T	R	P	S
T	0	0	0.11	0.89
R	0.03	0.97	0	0
P	0	0	0	1
S	0	1	0	0

TREATMENT 2 (A and B)

OPPONENT

	Matrix	C	D
SUBJECT	C	1pellet	8second
	D	3pellet	4second

OCCUPANCY STATE rate per session of R (mutual cooperation)

Sessions	1	2	3	4	5
Rat 1	0.80	0.93	0.70	0.83	0.70
Rat 2	0.13	0.47	0.20	0.10	0.28
Rat 3	0.80	0.63	0.87	0.73	0.77
Rat 4	0.93	0.73	0.67	0.90	1.00
Rat 5	0.27	0.23	0.53	0.21	0.27
Rat 6	0.40	0.47	0.53	0.53	0.53

OCCUPANCY STATE rate per session of S (sucker state)

Sessions	1	2	3	4	5
Rat 1	0.07	0.03	0.13	0.07	0.13
Rat 2	0.37	0.13	0.33	0.28	0.24
Rat 3	0.07	0.17	0.07	0.10	0.10
Rat 4	0.03	0.10	0.13	0.03	0.00
Rat 5	0.17	0.23	0.23	0.28	0.20
Rat 6	0.20	0.17	0.17	0.17	0.13

OCCUPANCY STATE rate per session of T (temptation state)

Sessions	1	2	3	4	5
Rat 1	0.07	0.03	0.13	0.07	0.13
Rat 2	0.37	0.17	0.33	0.31	0.28
Rat 3	0.10	0.17	0.07	0.10	0.10
Rat 4	0.03	0.10	0.13	0.03	0.00
Rat 5	0.17	0.27	0.23	0.28	0.23
Rat 6	0.20	0.17	0.17	0.17	0.17

OCCUPANCY STATE rate per session of P (Punishment state)

Sessions	1	2	3	4	5
Rat 1	0.07	0.00	0.03	0.03	0.03
Rat 2	0.13	0.23	0.13	0.31	0.21
Rat 3	0.03	0.03	0.00	0.07	0.03
Rat 4	0.00	0.07	0.07	0.03	0.00
Rat 5	0.40	0.27	0.00	0.24	0.30
Rat 6	0.20	0.20	0.13	0.13	0.17

Number of COOPERATION option in a session

Sessions	Rat 1	Rat 2	Rat 3	Rat 4	Rat 5	Rat 6
1	26	15	26	29	13	18
2	28	18	24	25	14	19
3	25	16	28	24	23	21
4	26	11	25	28	14	21
5	25	15	26	30	14	20

Markov Chain Matrix for each rat

Rat 1	T	R	P	S
T	0.00	0.00	0.38	0.62
R	0.10	0.90	0.00	0.00
P	0.00	0.00	0.00	1.00
S	0.17	0.83	0.00	0.00

Rat 2	T	R	P	S
T	0.00	0.00	0.40	0.60
R	0.54	0.46	0.00	0.00
P	0.00	0.00	0.48	0.52
S	0.55	0.45	0.00	0.00

Rat 3	T	R	P	S
T	0.00	0.00	0.33	0.67
R	0.15	0.85	0.00	0.00
P	0.00	0.00	0.00	1.00
S	0.00	1.00	0.00	0.00

Rat 4	T	R	P	S
T	0.00	0.00	0.33	0.67
R	0.07	0.93	0.00	0.00
P	0.00	0.00	0.40	0.60
S	0.00	1.00	0.00	0.00

Rat 5	T	R	P	S
T	0.00	0.00	0.44	0.56
R	0.48	0.52	0.00	0.00
P	0.00	0.00	0.60	0.40
S	0.42	0.58	0.00	0.00

Rat 6	T	R	P	S
T	0.00	0.00	0.76	0.24
R	0.33	0.67	0.00	0.00
P	0.00	0.00	0.24	0.76
S	0.08	0.92	0.00	0.00

REWARD: rate of maximum food in the game

Sessions	Rat 1	Rat 2	Rat 3	Rat 4	Rat 5	Rat 6
1	0.67	0.82	0.73	0.69	0.51	0.67
2	0.69	0.64	0.76	0.69	0.69	0.64
3	0.73	0.80	0.71	0.71	0.82	0.69
4	0.69	0.69	0.69	0.67	0.69	0.69
5	0.73	0.74	0.71	0.67	0.64	0.69

TIMEOUT accumulated in a session (Punish + ITI)

Sessions	Rat 1	Rat 2	Rat 3	Rat 4	Rat 5	Rat 6
1	0.20	0.87	0.17	0.07	0.73	0.60
2	0.07	0.50	0.37	0.27	0.73	0.53
3	0.30	0.80	0.13	0.33	0.47	0.47
4	0.17	0.86	0.27	0.10	0.79	0.47
5	0.30	0.69	0.23	0.00	0.70	0.43

TREATMENT 3 (A)

OPPONENT

	Matrix	C	D
SUBJECT	C	1pellet	8second
	D	5pellet	4second

OCCUPANCY STATE rate per session of R (mutual cooperation)

Sessions	1	2	3	4	5
Rat 1	0.37	0.13	0.10	0.10	0.37
Rat 2	0.76	0.70	0.68	0.53	0.70
Rat 3	0.53	0.27	0.37	0.43	0.53

OCCUPANCY STATE rate per session of S (sucker state)

Sessions	1	2	3	4	5
Rat 1	0.23	0.27	0.13	0.30	0.13
Rat 2	0.10	0.10	0.14	0.17	0.10
Rat 3	0.13	0.17	0.23	0.17	0.20

OCCUPANCY STATE rate per session of T (temptation state)

Sessions	1	2	3	4	5
Rat 1	0.23	0.27	0.17	0.30	0.17
Rat 2	0.10	0.13	0.14	0.20	0.13
Rat 3	0.13	0.17	0.23	0.17	0.20

OCCUPANCY STATE rate per session of P (Punishment state)

Sessions	1	2	3	4	5
Rat 1	0.17	0.33	0.60	0.30	0.33
Rat 2	0.03	0.07	0.04	0.10	0.07
Rat 3	0.20	0.40	0.17	0.23	0.07

Number of COOPERATION option in a session

Sessions	Rat 1	Rat 2	Rat 3
1	18	25	20
2	12	24	13
3	7	23	18
4	12	21	18
5	15	24	22

Markov Chain Matrix for each rat

Rat 1	T	R	P	S
T	0.00	0.00	0.61	0.39
R	0.56	0.44	0.00	0.00
P	0.00	0.00	0.56	0.44
S	0.45	0.55	0.00	0.00

Rat 2	T	R	P	S
T	0.00	0.00	0.47	0.53
R	0.21	0.79	0.00	0.00
P	0.00	0.00	0.00	1.00
S	0.00	1.00	0.00	0.00

Rat 3	T	R	P	S
T	0.00	0.00	0.58	0.42
R	0.37	0.63	0.00	0.00
P	0.00	0.00	0.42	0.58
S	0.16	0.84	0.00	0.00

REWARD: rate of maximum food in the game

Sessions	Rat 1	Rat 2	Rat 3
1	0.61	0.51	0.48
2	0.59	0.55	0.44
3	0.37	0.56	0.61
4	0.64	0.61	0.51
5	0.48	0.55	0.61

TIMEOUT: ratio of maximum timeout in a session

Sessions	Rat 1	Rat 2	Rat 3
1	0.63	0.24	0.47
2	0.87	0.27	0.73
3	0.87	0.32	0.63
4	0.90	0.43	0.57
5	0.60	0.27	0.47

EXPERIMENTE 3 (B)

OPPONENT

Matrix		C	D
SUBJECT	C	2pellet	8second
	D	3pellet	4second

OCCUPANCY STATE rate per session of R (mutual cooperation)

Sessions	1	2	3	4	5
Rat 1	0.43	0.33	0.50	0.47	0.40
Rat 2	0.37	0.07	0.27	0.27	0.30
Rat 3	0.50	0.53	0.63	0.73	0.55

OCCUPANCY STATE rate per session of S (sucker state)

Sessions	1	2	3	4	5
Rat 1	0.23	0.30	0.20	0.23	0.23
Rat 2	0.23	0.33	0.23	0.20	0.23
Rat 3	0.17	0.20	0.13	0.13	0.17

OCCUPANCY STATE rate per session of T (temptation state)

Sessions	1	2	3	4	5
Rat 1	0.27	0.30	0.20	0.23	0.23
Rat 2	0.27	0.33	0.27	0.20	0.27
Rat 3	0.17	0.20	0.13	0.13	0.17

OCCUPANCY STATE rate per session of P (Punishment state)

Sessions	1	2	3	4	5
Rat 1	0.07	0.07	0.10	0.07	0.13
Rat 2	0.13	0.27	0.23	0.33	0.20
Rat 3	0.13	0.07	0.10	0.00	0.10

Number of COOPERATION option in a session

Sessions	Rat 1	Rat 2	Rat 3
1	20	18	20
2	19	12	22
3	21	15	23
4	21	14	26
5	19	16	21

Markov Chain Matrix for each rat

Rat 1	T	R	P	S
T	0.00	0.00	0.22	0.78
R	0.44	0.56	0.00	0.00
P	0.00	0.00	0.33	0.67
S	0.26	0.74	0.00	0.00

Rat 2	T	R	P	S
T	0.00	0.00	0.47	0.53
R	0.37	0.63	0.00	0.00
P	0.00	0.00	0.49	0.51
S	0.65	0.35	0.00	0.00

Rat 3	T	R	P	S
T	0.00	0.00	0.45	0.55
R	0.25	0.75	0.00	0.00
P	0.00	0.00	0.08	0.92
S	0.08	0.92	0.00	0.00

REWARD: rate of maximum food in the game

Sessions	Rat 1	Rat 2	Rat 3
1	0.83	0.77	0.75
2	0.78	0.57	0.83
3	0.80	0.67	0.83
4	0.82	0.57	0.93
5	0.75	0.70	0.81

TIMEOUT accumulated in a session (Punish + ITI)

Sessions	Rat 1	Rat 2	Rat 3
1	0.53	0.60	0.43
2	0.67	0.93	0.47
3	0.50	0.70	0.37
4	0.53	0.73	0.27
5	0.60	0.67	0.45

EXPERIMENTE 1 – REVERSION

		OPPONENT	
	**Matrix**	C	D
SUBJECT	C	1pellet	8second
	D	2pellet	4second

Number of COOPERATION option in a session

Sessions	Rat 1	Rat 2	Rat 3	Rat 4
1	0.63	0.90	0.83	0.76
2	0.70	0.90	0.93	1.00
3	0.90	0.93	1.00	1.00
4	0.83	0.86	0.83	1.00
5	0.87	0.80	0.69	1.00
6	0.80	0.93	0.90	1.00
7	0.80	0.83	0.77	0.97
8	0.73	0.72	0.80	1.00
9	0.83	0.86	0.83	1.00
10	0.83	0.97	0.86	1.00

REWARD: rate of maximum food in the game

Sessions	Rat 1	Rat 2	Rat 3	Rat 4
1	0.90	1.00	0.97	0.73
2	0.93	1.00	1.00	1.00
3	1.00	1.00	1.00	1.00
4	0.97	0.97	0.93	1.00
5	1.03	0.97	0.93	1.00
6	1.00	1.00	0.97	1.00
7	1.03	0.97	0.93	1.00
8	0.97	0.97	0.97	1.00
9	0.97	1.00	0.97	1.00
10	1.00	1.00	1.00	1.00
